# Supplementary material for: Microglial CD74 Expression Is Regulated by TGFβ Signaling
Source: Int J Mol Sci. 2022 Sep 6;23(18):10247. doi: 10.3390/ijms231810247 (PMC9499470; doi:10.3390/ijms231810247)
Supplement: Supplementary file 1 [file ijms-23-10247-s001.zip › ijms-1894034-supplementary.pdf]

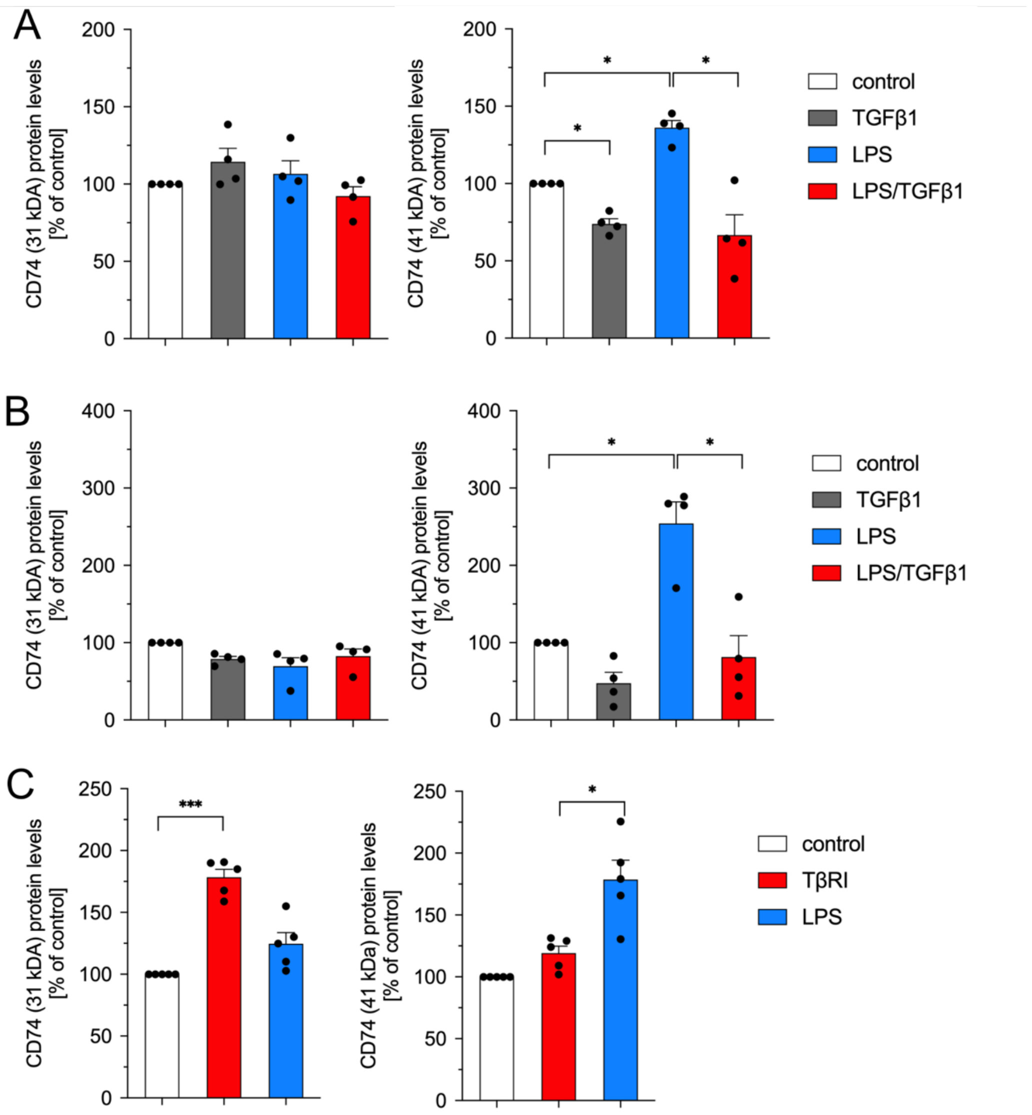

Figure S1. Densitometric analysis of 31 kDa and 41 kDa isoform levels in primary microglia. TGFβ1 inhibits LPS-mediated upregulation of Cd74 41 kDA isoform in primary microglia. Levels of 31 kDa and 41 kDa isoforms after treatment of microglia with TGFβ1 (5 ng/ml), LPS (1 µg/ml) or both factors for 6 h (**A**) and 24 h (**B**). CD74 isoform levels after treatment of mixed glia cultures with TβRI (500 nM) or LPS (1 µg/ml) for 24 h (**C**). Data are given as means ± SEM for at least four independent experiments. *P*-values derived from one-way ANOVA followed by Tukey´s multiple comparison tests are **p*<0.05 and ****p*<0.001.
